# Recovery from supercooling, freezing, and cryopreservation stress in larvae of the drosophilid fly, *Chymomyza costata*

**DOI:** 10.1038/s41598-018-22757-0

**Published:** 2018-03-13

**Authors:** Tomáš Štětina, Petr Hůla, Martin Moos, Petr Šimek, Petr Šmilauer, Vladimír Koštál

**Affiliations:** 1 Faculty of Science, University of South Bohemia, Branišovská 31, 37005 České Budějovice, Czech Republic; 2 Biology Centre, Czech Academy of Sciences, Institute of Entomology, Branišovská 31, 37005 České Budějovice, Czech Republic

## Abstract

Physiological adjustments accompanying insect cold acclimation prior to cold stress have been relatively well explored. In contrast, recovery from cold stress received much less attention. Here we report on recovery of drosophilid fly larvae (*Chymomyza costata)* from three different levels of cold stress: supercooling to −10 °C, freezing at −30 °C, and cryopreservation at −196 °C. Analysis of larval CO_2_ production suggested that recovery from all three cold stresses requires access to additional energy reserves to support cold-injury repair processes. Metabolomic profiling (targeting 41 metabolites using mass spectrometry) and custom microarray analysis (targeting 1,124 candidate mRNA sequences) indicated that additional energy was needed to: clear by-products of anaerobic metabolism, deal with oxidative stress, re-fold partially denatured proteins, and remove damaged proteins, complexes and/or organelles. Metabolomic and transcriptomic recovery profiles were closely similar in supercooled and frozen larvae, most of which successfully repaired the cold injury and metamorphosed into adults. In contrast, the majority of cryopreseved larvae failed to proceed in ontogenesis, showed specific metabolic perturbations suggesting impaired mitochondrial function, and failed to up-regulate a set of 116 specific genes potentially linked to repair of cold injury.

## Introduction

Insects evolved complex and efficient strategies for survival at body temperatures below the equilibrium melting point of their body liquids^1,2^. The insect cold tolerance literature has primarily focused on physiological mechanisms that accompany seasonal or rapid cold hardening and help to prevent occurrence of cold injury^3^. Thus, cold hardening has been associated with global changes in gene transcription, protein expression, and metabolome composition^4^; transition from active life to developmental arrest called diapause^5^; accumulation of low-molecular weight cryoprotectants^6^; synthesis of proteins which regulate the process of ice formation^7^; compositional remodeling of cell membranes^8^; and rearrangement of cytoskeleton structure^9,10^. Adapted and properly acclimated cold hardy insects are often *a priori* considered to be resistant to the occurence of cold injury. Nevertheless, their cold hardiness might also be based, at least partly, on their abilty to tolerate or even actively repair the injury that potentially incurred during the cold stress.

Participation of active repair processes in recovery from cold stress is supported by observations of cold-induced upregulation of the activity of cellular protective systems preventing apoptosis^11^, oxidative damage^12,13^, and loss of proteins’ native conformation^14^. The activation of heat shock protein (HSP) production is the most typical immediate physiological response to environmental stress observed in almost all organisms^15^ including insects exposed to cold^16–23^. HSPs are ubiquitous molecular chaperones that can prevent the irreversible aggregation of cold-denaturing proteins in an ATP-independent manner^24^, or assist in protein re-folding and protein cellular degradation in an ATP-dependent manner^25^. At the insect survival level, the participation of active repair processes in recovery from cold stress is supported by observation of ‘delayed mortality’ – a mortality which does not occur immediately upon cold stress but instead later during ontogenesis^26–28^. Though delayed mortality is considered as critically important for ecologically meaningful interpretation of survival assays^29,30^, it is often neglected in experimental practice because of costs linked with maintaining the insect culture for long periods of time following treatment^29^. The delayed mortality might be, in theory, regarded as an inability to repair any vital injury that occurred, or was triggered, during previous exposure to cold. Additionally, the cost of cold-injury repair processes may be manifested as a reduction in fitness for survivors (i.e. offspring production), which has also previously been demonstrated in some studies^31–33^. We argue that additional studies on recovery from cold stress are greatly needed in order to understand the whole process of insect cold tolerance.

Here, we report on recovery from cold stress in the larvae of drosophilid fly, *Chymomyza costata*. We used diapausing larvae acclimated to a relatively low temperature of 4 °C because such larvae are physiologically ‘uniform’ and exhibit the highest level of cold hardiness^34^. We exposed the larvae to one of three different levels of cold stress: supercooling to −10 °C (S), freezing at −30 °C (F), or cryopreservation in liquid nitrogen at −196 °C (LN), and observed changes in their metabolomic and transcriptomic profiles for three days of recovery from cold stress, at 18 °C. Transferring the control, non-stressed larvae (C) to 18 °C was used to subtract the responses linked to resumption of locomotion, feeding, metabolic activity, and continuation in ontogenesis toward pupation. In addition, we estimated the rate of resumption of larval activity upon transfer to 18 °C by analyzing how they balance cold-induced hyperkalemia (via measurement of hemolymph potassium concentrations) and how they increase their metabolic rate (via monitoring of CO_2_ production). We expected that increasing the dose of cold stress (S < F < LN) will increase the proportion of delayed mortality in *C*. *costata* larvae, as different stresses may cause quantitatively and/or qualitatively different injuries. Specifically, we aimed to obtain insight into the nature of cold injuries and their repair based on metabolomic and transcriptomic profiling. We hypothesized that: (I) The metabolomic and transcriptomic profiles will be similar in C and S larvae, as there is almost no delayed mortality in these variants (i.e. supercooling does not seem to cause any injury and requires no repair). (II) The metabolomic and transcriptomic profiles will be different between S and F larvae, as the conditions of S and F treatments widely differ. The F treatment includes growth of extracellular ice crystals, freeze-induced cellular dehydration, and shrinkage associated with a whole array of deleterious consequences^35^, while the S larvae experience ‘only’ a decrease of temperature; the cell volume, water activity, and associated parameters remain relatively stable. (III) The metabolomic and transcriptomic profiles will be similar in F and LN larvae, as they experience similar (high) magnitude of freeze-induced dehydration. The potential differences between F and LN larvae will help to identify structures and processes vulnerable to injury caused by rapid changes of temperature during cryopreservation.

## Results and Discussion

### Larvae are not instantaneously killed by cold stress but may die later in ontogenesis

Survival of larvae, checked 12 h after the transfer to 18 °C, was relatively high in all experimental variants (control and three different cold-stress treatments), ranging between 84.5% and 100% (Table 1). Larvae pupariated on average 18.7 days after the transfer to 18 °C, and another 12.2 days were required for pupal metamorphosis and adult emergence (Table 1). Some individuals perished later during the development, many days or even weeks after the end of cold stress. This delayed mortality was low or practically absent in the C, S, and F experimental variants (where 93.6, 95.0 and 89.3% of larvae survived to adult stage, respectively). In contrast, delayed mortality was relatively high in cryopreserved larvae (LN), where only 39.5% of larvae were able to metamorphose into adults (Table 1).

**Table 1:** Survival of *Chymomyza costata* larvae after different cold stresses.

Parameter	Units	Sex	Control (C)	Cold stress		
				Supercooling (S)	Freezing (F)	Cryopreservation (LN)
**Survival analysis**						
Survival*, larva, 12 h	%	n.a.	100 (272)	99.2 (200)	100 (300)	84.5 (200)
Survival, puparium	%	n.a.	97.3	99.0	95.7	48
*Time to pupariation*	*days*	n.a.	*18*.*7* ± *0*.*4 (14*.*0–25*.*2)*	n.a.	n.a.	n.a.
Survival, adult	%	n.a.	93.6	95.0	89.3	39.5
*Time to adult emergence*	*days*	n.a.	*30*.*9* ± *0*.*03 (27*.*1–38*.*0)*	n.a.	n.a.	n.a.

*Numbers of larvae used for survival analysis (*n*) are shown in parentheses.

Data on developmental timing (in *italics*) are shown as mean ± S.D.; in addition, the range is shown in parentheses for developmental timing.

n.a., not analyzed.

### Recovery from cold stress requires energy

We observed that almost all cold-stressed larvae were able to restore their locomotor activity relatively rapidly upon transfer to 18 °C. In fact, many larvae (irrespective of treatment) actively crawled in a fully coordinated way already within the period of handling after the end of cold stress, prior to the start of physiological measurements. Restoration of locomotor behavior requires coordinated neuromuscular activity, which in turn depends on normal (i.e. uneven) distribution of ions across biological membranes. It is a well-known fact that cold exposure dissipates transmembrane electrochemical potentials in non-adapted and/or non-acclimated animals^36,37^. Adapted and cold-acclimated ectotherms, however, maintain transmembrane electrochemical potentials when chilled or even supercooled for long periods of time, which is regarded as one of the crucial physiological mechanisms supporting their cold tolerance^38–40^. In freeze-tolerant insects, however, the transmembrane electrochemical potentials dissipate during freezing and are restored rapidly upon melting^41^. Hemolymph hyperkalemia (increasing [K^+^]) is thus a typical sign of disturbed ionic regulation both during insect freezing^41^ and lethal supercooling^40,42–44^.

**Figure 1:**
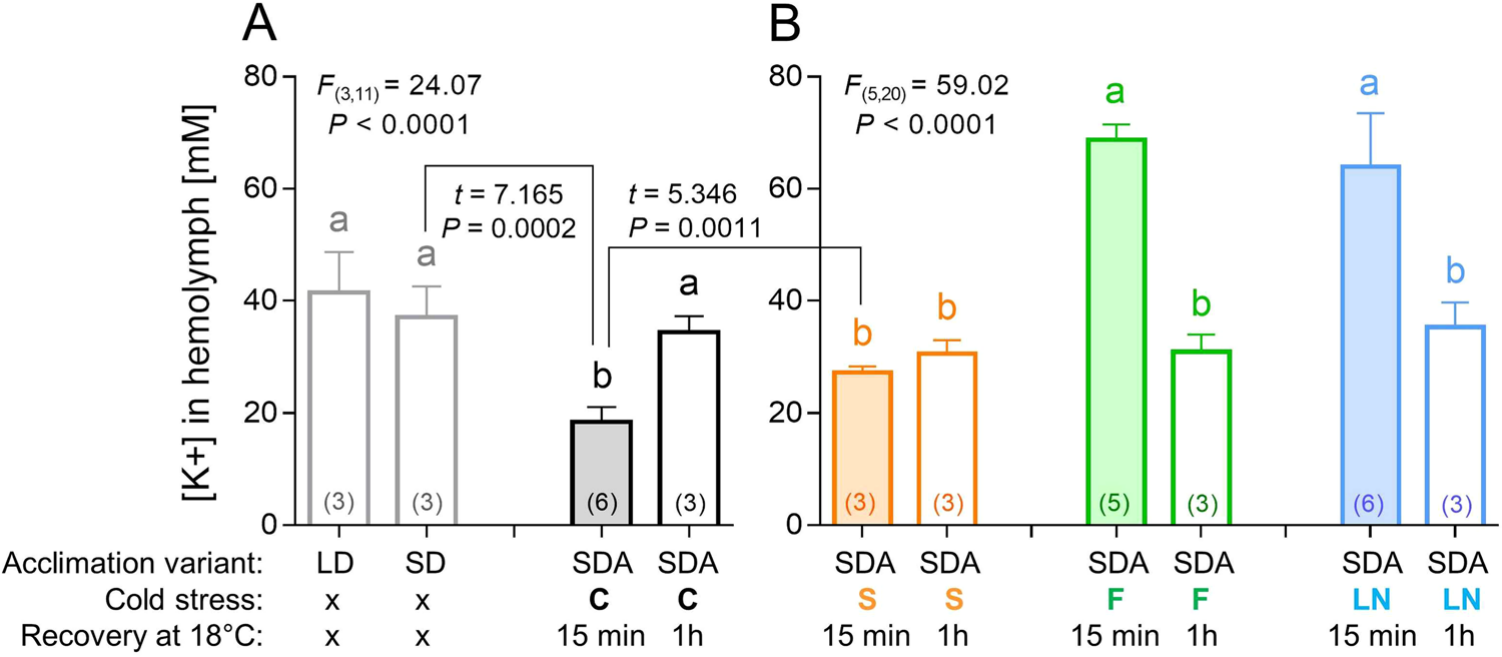
Hemolymph potassium concentration in control and cold-stressed *Chymomyza costata* larvae. Each column shows the mean (and S.D.) of [K^+^] analyzed using an ion-selective K^+^ microelectrode in a sample of hemolymph collected from a pool of 10–20 larvae (the number of pools, i.e. biological replicates are shown in parentheses). The larvae were variously acclimated: LD, fully active and developing, warm-acclimated; SD, diapausing, warm-acclimated; and SDA, diapausing, cold-acclimated. (**A**) The control larvae, C, were not subjected to cold stress but were transferred from 4 °C directly to 18 °C and the [K^+^] was measured 15 min or 1 h after the transfer. (**B**) The cold-stressed larvae were: S, supercooled to −10 °C; F, frozen at −30 °C; or LN, cryopreserved in liquid nitrogen. After the cold stress, the larvae were transferred to 18 °C and the [K^+^] was measured 15 min or 1 h after the transfer. Differences between treatments were analyzed using ANOVA followed by Bonferroni’s test (columns flanked by different letters are statistically different). The control treatments (**A**) and cold-stressed treatments (**B**) were analyzed separately and *F* statistics and *P* values are presented. In addition, t-tests were used to compare two specific treatments (connected by zig-zag lines) and *t* statistics and *P* values are shown. See text for more details.

We found [K^+^] of 41.5 mM in hemolymph of active, directly developing larvae of *C*. *costata* (acclimation variant LD; for explanation of acclimation variants, see Fig. S1). A similar value (37.1 mM) was seen in warm-acclimated, diapausing larvae (acclimation variant SD). The cold-acclimated diapausing larvae (acclimation variant SDA) exhibited significantly lower [K^+^] of 18.5 mM but it rapidly increased (within 1 h) to 34.4 mM upon transfer to 18 °C (Fig. 1A). In theory, the relatively low [K^+^] in SDA larvae might have an adaptive meaning as it would help to counteract hyperkalemia during exposures to cold extremes^45^. Supercooled larvae (S) displayed a [K^+^] corresponding to untreated controls. In contrast, frozen (F) and cryopreserved (LN) larvae showed significantly higher [K^+^] when measured 15 min after the end of cold stress: 68.7 mM and 64.0 mM, respectively. These results demonstrate that larval freezing is associated with dissipation of transmembrane electrochemical potentials, similarly as it was shown previously in frozen larvae of the fly, *Xylophagus cincta*^41^. At 1 h of recovery, the larvae of all treatments showed similar [K^+^]: 30.6 mM (S); 31.0 mM (F); and 35.3 mM (LN), which is close to a value observed in 18 °C-exposed controls (Fig. 1B). These results show that frozen larvae were able to fully restore dissipated electrochemical potentials within 1 h after the cold stress. Such exercise undoubtedly required energy supply for the activity of primary ion pumping ATPases, which establish ionic homeostasis.

**Figure 2:**
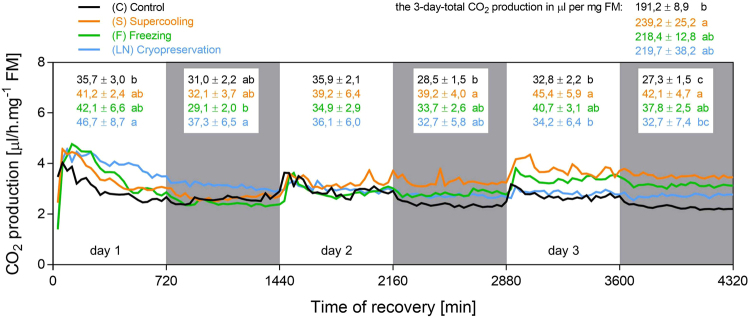
CO_2_ production in control and cold-stressed *Chymomyza costata* larvae. Each line represents a mean record of CO_2_ production analyzed in six groups of ten larvae. The record was taken in 30 min intervals during three days (4,320 min) of recovery at 18 °C from different cold stresses (S, F, LN, see Fig. 1 and text for more details). The control larvae (C) were not subjected to cold stress but were transferred from 4 °C directly to 18 °C. White and grey areas represent alteration of light and dark conditions (12 h L, 12 h D). The numbers show mean ± S.D. CO_2_ production is expressed in µl per mg fresh mass (FM) for each experimental variant (color coded) during 12 h intervals and also during the whole three day period (means flanked by different letters are statistically different according to ANOVA followed by Bonferroni’s test). See Dataset S1 for more details.

Next, we measured CO_2_ production as a direct proxy of metabolic rate and energy turnover during recovery from cold stress (Fig. 2, see Dataset S1 for more details). In all experimental variants, handling the larvae prior to the start of measurement took approximately 30 min during which no data were taken. During the first 30-min interval of measurement (30–60 min after cold stress), the values of CO_2_ production (µl of CO_2_ produced per mg FM per interval) significantly differed among the variants: 1.73 ± 0.42 (C); 1.23 ± 0.35 (S); 0.70 ± 0.45 (F), 0.75 ± 0.84 (LN) (ANOVA *F*_(3, 20)_ = 4.683; *P = *0.0123), and Bonferroni’s multiple comparisons post hoc test found significant differences between C vs. F and also C vs. LN variants. These results document that larvae which underwent freezing event (F and LN) were recovering slightly slower than control larvae (C). The values of CO_2_ production were similar in all variants within the second interval of measurement (60–90 min after cold stress) ranging between 1.99 and 2.29 µl CO_2_ (ANOVA *F*_(3, 20)_ = 0.7073; *P* = 0.5589). These results confirm that larval recovery from cold stress is relatively fast in all treatments. An apparent overshoot of CO_2_ production was detectable during first hours of recovery in all treatments. The overshoot was most probably associated with high locomotion activity stimulated by handling and transfer to light and 18 °C (we observed that larvae vigorously crawled and climbed the walls of rearing tubes during the first hours after the transfer).

Figure 2 suggests that there were no profound differences among four experimental variants in the overall pattern of CO_2_ production over 3 days. Nevertheless, after summing up the total production of CO_2_ over the whole 3 day period, all three treatments showed higher values than control. A difference of 48 µl.mg^−1^ FM of CO_2_, as found between S and C larvae, was statistically significant (239.2 vs. 191.2 µl.mg^−1^ FM of CO_2_, respectively). A typical SDA larva has a FM of approximately 2 mg and carries approximately 40–50 µg of glycogen and 250–300 µg of total lipids^34^. In theory, one larva would need to oxidize 80 µg of carbohydrate (glycogen) or 140 µg of lipidic energy substrate (calculated using the coefficients by Schmidt-Nielsen^46^) to produce 2 × 48 = 96 µl of CO_2_ (per 2 mg of FM). This calculation suggests that considerable part of energy turnover (approximately 20%) in a larva recovering from cold stress is devoted to the repair processes. This is agreement with other studies^31–33^ that indirectly demonstrate the existence of a cost of cold-injury repair processes in the form of impaired fitness of survivors (reduced reproductive capacity). It seems as counter-intuitive that the cost of repair processes was highest in S larvae while it was lower in F and LN larvae that experienced ‘harsher’ treatments. We can offer a speculative explanation that an unknown proportion of living (during analysis) but doomed larvae in F and (especially) LN treatments (see Table 1) might cause an underestimation of the true cost of repair after the freezing and cryopreservation stress. Collectively, our data and the literature suggest that insect recovery from cold stress requires access to energy, which is used to re-establish the homeostasis at warm conditions and to repair cold-induced injuries.

In addition, we analyzed the patterns of CO_2_ production in order to detect presence of daily rhythmicity and any potential differences in this parameter. Visual inspection of the patterns on Fig. 2 suggests that larvae exhibit only very weak daily rhythmicity in CO_2_ production at best. A small difference between day and night CO_2_ production rates is visually detectable in all experimental variants. The presence of a peak in CO_2_ production stimulated by light ON is also clearly detectable in all variants. Results of statistical analysis of daily rhythmicity are presented in the supplementary information (Dataset S1). Based on these results, we can conclude that the cold stress did not interfere with neuromuscular responsiveness of *C*. *costata* to a ‘morning’ light stimulus.

### Metabolite profiling revealed themes common to all treatments but also separated the LN vs. S and F treatments

Results of targeted metabolomics are summarized in Dataset S2. As demonstrated in Fig. S1 (Dataset S2, Excel sheet: Results), the sum concentrations of amino-compounds, sugars and polyols, and organic acids (intermediates of glycolysis and TCA cycle) remained relatively stable and did not differ substantially among the experimental variants. Four of five principal metabolites (those which together represent > 87% of the total concentration of all 41 analyzed metabolites), namely proline, glutamine, trehalose, and asparagine, showed similar temporal profiles in all four variants (Fig. S2, Dataset S2, Excel sheet: Results). The fifth principal metabolite, alanine, will be discussed later.

**Figure 3:**
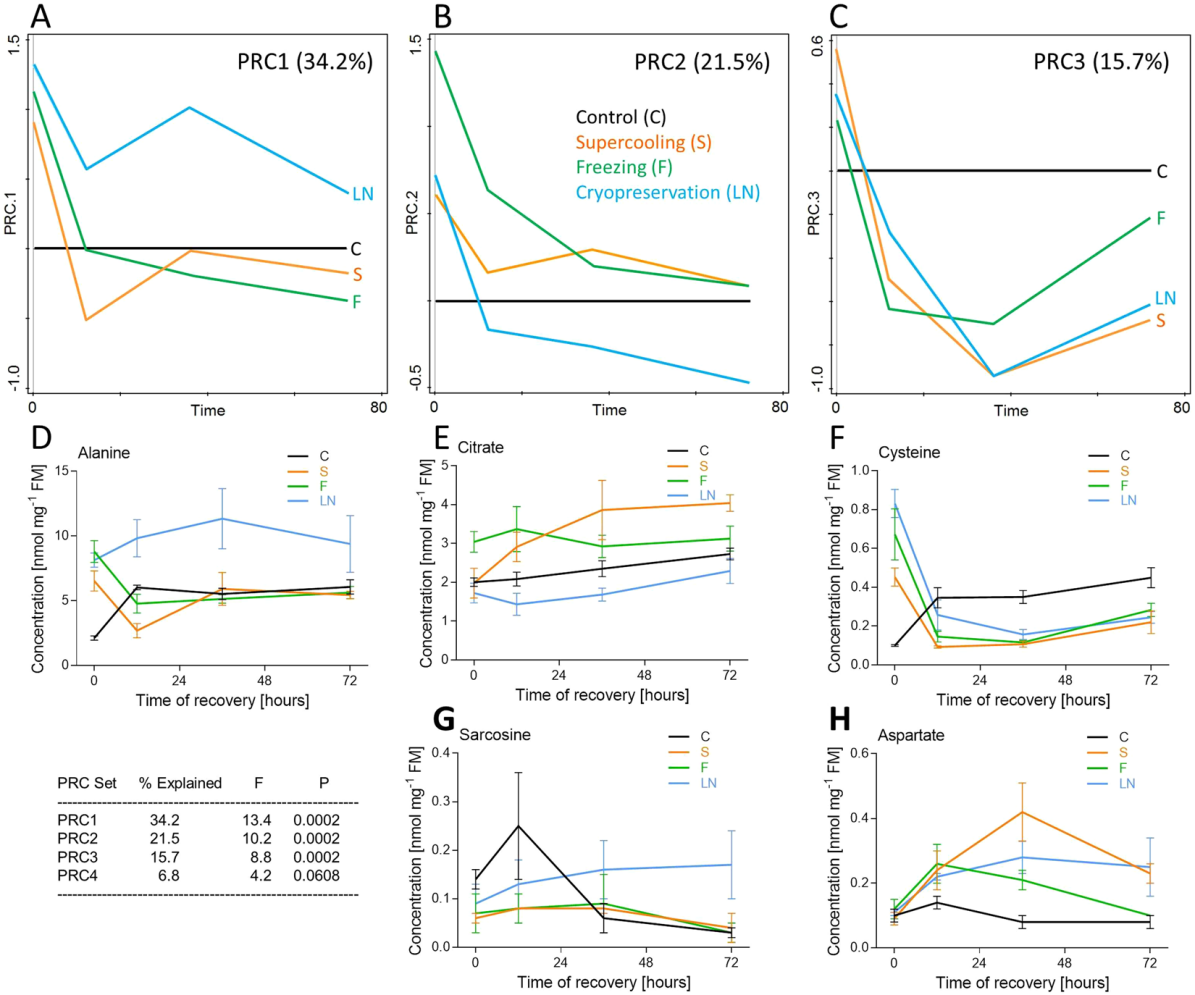
Metabolite profiles of control and cold-stressed *Chymomyza costata* larvae. The targeted metabolomics of 41 different metabolites was conducted using a combination of mass spectrometry-based analytical methods. (**A–C**) The principal response curve (PRC) model found three principal PRC sets showing statistically significant proportion of variation explained by the main effect of treatment plus its interaction with time. In control larvae (C), the temporal pattern of metabolic changes during their 3-day recovery after transfer to 18 °C is levelled to 0 and the temporal patterns of three cold-stressed treatments (S, F, LN) are normalized to the control. (**D–H**) Examples of real concentration changes during recovery from cold stress in selected best-predicted metabolites are shown for each PRC set (observe columns below PRCs 1, 2, 3). See text and Dataset S2 for more details.

Statistical analysis (PRC) was used to detect leading patterns in temporal metabolomic profiles and, mainly, to identify where and how these patterns differ among treatments. The proportion of explained variation for a single particular metabolite exceeded our arbitrary threshold value Cfit > 0.5 in only two cases (alanine and fructose in PRC set 1). The magnitudes of responses were relatively low, i.e. exceeding the arbitrary threshold value of Resp >  ± 2.0 in only three cases (alanine in PRC set 1, and cysteine and aspartate in PRC set 3), which led us to consider a less stringent threshold value of Resp >  ± 1.5. Applying this less-stringent threshold, 18 metabolites (37.5% of 41 targeted metabolites) exhibited temporal patterns of concentration changes during recovery deviating prominently between treated (S, F, and LN) vs. control (C) larvae (Dataset S2, Excel sheet: PRC analysis). The PRC analysis thus revealed that the recovery from cold stress (irrespective of the treatment) differs from a mere resumption of larval activities upon transfer from low to high temperature. The PRC model had three PRC sets showing statistically significant proportion of variation explained by the main effect of treatment plus its interaction with time (Fig. 3A–C). The fourth PRC set, though also marginally statistically significant, is not presented in Fig. 3 as the proportion of explained variation (6.8%) was relatively low. For each PRC set, we present examples of the best-predicted metabolites according to a combination of Resp and Cfit parameters (Fig. 3D–H).

The PRC set 1 (Fig. 3A) shows a difference in temporal patterns between LN vs. S and F treatments. This difference was best represented by alanine (Fig. 3D), fructose and several other metabolites (for details, see Dataset S2, Excel sheet: PRC analysis). At time 0, their concentrations were higher than control in all three treatments. Later during recovery, however, the concentrations either dropped below or close to control values (S and F) or remained relatively high or even increased (LN). The PRC set 2 (Fig. 3B) detected, again, a difference between LN vs. S and F treatments, which was represented best by citrate (Fig. 3E), ketoglutarate, and some other metabolites on one side (pattern PRC2a) vs. sarcosine (Fig. 3G) on the other side (pattern PRC2b). In the pattern PRC2a, initially high levels of a metabolite decreased during recovery but either remained above control (S and F) or dropped below control (LN). The pattern PRC2b was opposite: initially low levels increased during recovery but either remained close to control (S and F) or increased significantly above control (LN). The PRC set 3 (Fig. 3C) separated the temporal profiles according to quantitative differences in concentrations, while the directions of responses (higher/lower than control) were similar in all treatments. The pattern PRC3a associated metabolites such as cysteine (Fig. 3F), succinate, and lactate, showing very high concentrations at time 0 followed by a rapid drop below the control levels during recovery. In contrast, the pattern PRC3b associated metabolites such as aspartate (Fig. 3H), glutamate, and glycine, showing higher concentrations in treatments than in control during recovery.

While interpreting the results of metabolomics, we noticed commonalities and differences among three different cold stress treatments. The strong commonality suggests that all three cold stresses exhibited partially overlapping effects on larval physiology (caused similar sort of cold injury) that were not observed in control larvae. The differences unexpectedly draw the main division line between S and F vs. LN rather than between supercooled (S) vs. frozen (F and LN) larvae.

#### Commonalities

The larvae of all cold treatments sampled at time 0 showed clear symptoms of a past anaerobic episode in their metabolism, such as high lactate, succinate, and alanine concentrations in comparison to control^47,48^. In addition, all cold-treated larvae showed high levels of cysteine at time 0, which might indicate a disturbance of redox homeostasis and oxidative stress^49,50^. During the next hours of recovery, anaerobic by-products were rapidly cleared (with a single exception: alanine in LN), and cysteine concentrations decreased below the control levels, indicating return to aerobic homeostasis. As another commonality, concentrations of aspartate and glutamate transiently increased during recovery in cold-treated variants in comparison to control. The aspartate and glutamate may serve as sinks for amino groups in transamination reactions during catabolism of all amino acids^51^. Therefore, transiently increasing levels of glutamate and aspartate might be associated with increasing rates of protein degradation which, in turn, may reflect higher rates of removal of cold-injured proteins and cold-induced chaperones^14,25^. As chaperoning requires ATP, this result indirectly supports our data on increased energy requirement during recovery from cold stress.

#### Differences

The metabolic perturbations observed specifically in the LN treatment seem to point toward impaired mitochondrial function during unsuccessful recovery/delayed mortality following cryopreservation: (i) The accumulations of glucose and several derivates of the glycolytic pathway side-branches, such as fructose, myo-inositol, sorbitol, and glycine, suggest that the glycolytic flux (liberated from glycogen and/or absorbed from diet) is partially diverted from TCA and production of energy toward accumulation of alanine^52^. (ii) The catabolism of lactate and cysteine, that were accumulated during the previous anaerobic episode, might contribute to production of excess pyruvate, which again might be canalized preferentially to alanine rather than to TCA. (iii) The level of sarcosine (a derivative of glycine) has been reported to increase in cancer cells, known to divert their glycolytic flux from TCA to anaerobic end-products^53^. (iv) The relatively low levels of citrate, aconitate, and ketoglutarate might result from partial blockade of pyruvate entry to, or early steps of, the TCA cycle in the mitochondrial matrix [see (i) – (iii)]^48^. (v) The accumulation of ornithine (the synthesis of which is also located inside mitochondria) might indicate re-routing of mitochondrial catabolic pathways for excess amino acids (proline, glutamine, glutamate) from TCA (partially blocked) toward ornithine^54^. At the same time, the polyamines (putrescine, spermine, and spermidine), to which ornithine can be further transformed upon decarboxylation in the cytosol, were undetectable or found in traces in all treatments. (vi) The last segment of the TCA cycle, catabolism of succinate^48^, that accumulated during a previous anaerobic episode, seems to operate normally in LN larvae.

### Gene transcript profiling revealed general transcriptional upregulation during recovery but again separated the LN vs. S and F treatments

**Figure 4:**
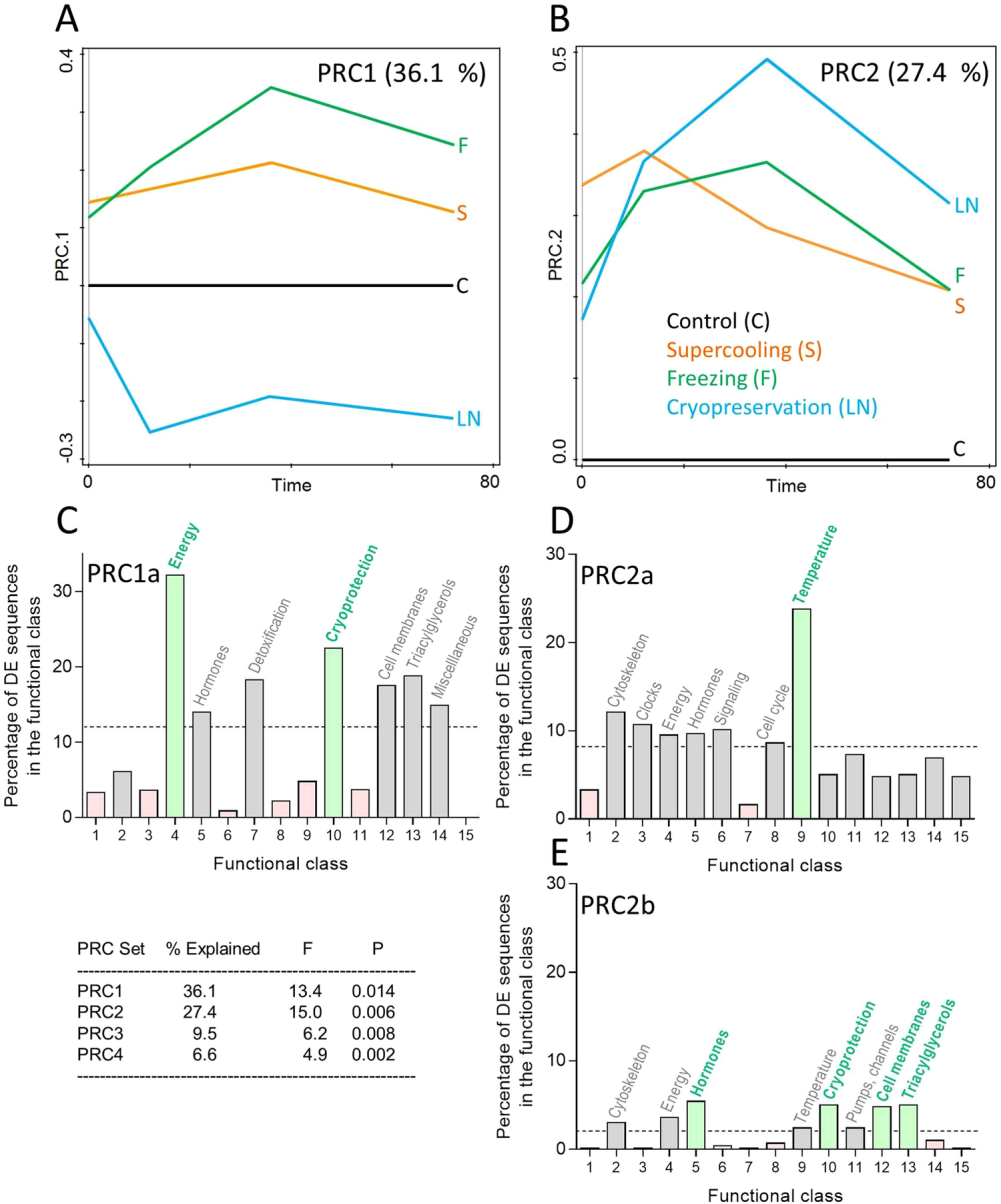
Gene transcript profiles of control and cold-stressed *Chymomyza costata* larvae. The transcriptomics analysis was conducted using custom C. costata microarrays containing probes for 1,124 candidate gene sequences. (**A**,**B**) The principal response curve (PRC) model found two principal PRC sets showing relatively high and statistically significant proportion of variation explained by the main effect of treatment plus its interaction with time. In control larvae, C, the temporal pattern of transcriptional changes during their 3-day recovery after transfer to 18 °C is levelled to 0 and the temporal patterns of three cold-stressed treatments (S, F, LN) are normalized to the control. (**C–E**) The differentially expressed (DE) sequences for each PRC set (1, 2) are clusterred in functional classes and percentages of DE genes within each functional class (enrichment) is scored. See Dataset S3 for calculation of the percentages of DE sequences and for more details.

Results of targeted transcriptomics are summarised in Dataset S2. According to PRC analysis, 223 differentially expressed (DE) sequences (19.8% of the 1,124 candidate sequences represented on the custom microarray) exhibited temporal patterns of expression during recovery deviating significantly between cold-treated (S, F, and LN) vs. control (C) larvae. Therefore, recovery from cold stress was clearly identified as an exercise differing in many aspects from a mere resumption of metabolic, behavioral, and developmental activities upon transfer of (control) larvae from low to high temperature. For filtering the DE sequences, we applied the criteria: Resp >  ± 2.0, and Cfit > 0.5 (Dataset S2, Excel sheet: Sequences and PRC analysis). The PRC model had two PRC sets showing statistically significant proportions of variation explained by the main effect of treatment plus its interaction with time (Fig. 4A,B). Two other PRC sets, PRC3 and PRC4, though also statistically significant, are not presented in Fig. 4 as the proportions of explained variation (9.5% and 4.4%, respectively) were relatively low.

The PRC set 1 (Fig. 4A) separated the LN treatment from the other two cold treatments (S and F) according to temporal patterns of 119 DE sequences. Most (116) of PRC1 DE sequences were upregulated during recovery in S and F treatments, but down-regulated (or not different from control) in the LN treatment (pattern PRC1a). Several examples of the predicted DE sequences are shown in Fig. S3 (Dataset S2, Excel sheet: PRC1 DE sequences). The Seq. 81398, *ornithine decarboxylase 1* (*odc1*) was the best-predicted PRC1 DE sequence (Cfit = 0.81; and Resp = 3.05), and the temporal patterns of its expression were validated by RT-qPCR method (Fig. S5, Dataset S2, Excel sheet: RT-qPCR validation). Only three PRC1 DE sequences showed an opposite pattern (PRC1b, not shown in figure): upregulated during recovery from LN treatment, while not different from control in S and F treatments (pattern PRC1b). The Seq. 60138, *heat shock protein 22* (*hsp 22*) was the best-predicted (Cfit = 0.42; and Resp = −3.34) of the three sequences (Fig. S3, Dataset S3, Excel sheet: PRC1 DE sequences; validation: Fig. S15, Dataset S3, Excel sheet: qRT-PCR validation). Looking at 116 PRC1a DE sequences according to functional categories, two classes emerged as most often represented (enriched classes): Energy and Cryoprotection (Fig. 4C).

The PRC set 2 (Fig. 4B) separated the treatments according to quantitative differences in sequence expression while the directions of responses (higher/lower than control) were similar in all treatments. The PRC2a pattern associated 94 sequences that were up-regulated in all three treatments during recovery in comparison to control. This pattern was dominated by a functional class Temperature (Fig. 4D) and the Seq. 53852, *centromere identifier* was the best-predicted example with Cfit = 0.86, and Resp = 5.05 (Fig. S4, Dataset 2, Excel sheet: PRC2 DE sequences; validation: Fig. S5, Dataset S3, Excel sheet: qRT-PCR validation). The PRC2b pattern associated 24 sequences that were down-regulated in all treatments during recovery in comparison to control. This pattern was driven by several functional classes (Fig. 4E) and the Seq. 3875, *histidine decarboxylase* was the best-predicted example with Cfit = 0.70, and Resp = −2.78 (Fig. S4, Dataset 2, Excel sheet: PRC2 DE sequences; validation: Fig. S5, Dataset S3, Excel sheet: qRT-PCR validation). Like in the metabolomics dataset, the transcriptomics dataset suggested that there are strong commonalities and some differences in the temporal patterns of gene expression during recovery from three different cold stresses.

#### Commonalities

Transcriptional upregulation dominated over downregulation during recovery. Both PRC sets 1 and 2 were driven mainly by DE sequences showing upregulation-type response in comparison to control (Fig. 4A,B and Figs S3 and S4). The upregulated DE sequences most often fall into three functional classes: Energy, Temperature, and Cryoprotection (Fig. 4C,D) which we interpret in a following way: As transcriptional upregulation generally consumes energy, the results support existence of energetic cost of repair processes. The repair processes might be linked with clearance of by-products of anaerobic metabolism, return of redox homeostasis, dealing with oxidative stress [all suggested by metabolomic profiling (Fig. 3) and also by literature^13,55,56^], and re-folding or removal of proteins that were partially denatured during cold stress [again reflected in metabolomic profiling (Fig. 3) and literature^22^]. The catabolism of putative cryoprotectants, such as proline, glutamine and trehalose, might differ (in terms of preferred pathways and/or rates of catabolism) between control and cold-treated larvae. The cryoprotectants were accumulated in large quantities during the long-term cold acclimation prior to cold stress^34^, and might serve as alternative energy substrates during subsequent ontogenesis^6,57^. Our metabolomics profiling, however, did not suggest any rapid clearance of cryoprotectants during the first three days of recovery from cold stress (Fig. S2). The elevated Cryoprotection DE transcripts, and the clearance of cryoprotectants, may thus come into effect only later during ontogenesis, as the larvae require 2–3 weeks for pupariation (Table 1).

#### Differences

We interpret the results of PRC set 1a as suggesting that LN larvae, in contrast to S and F larvae, failed to upregulate a number of sequences that might be important for successful repair of cold injury and averting the delayed mortality during recovery from cold stress. In the most enriched functional class, Energy (32.2% DE sequences, Fig. 4C), the whole spectrum of sequences coding for key enzymes of the main axis of intermediary and energy metabolism is represented, including: carbohydrate digestion (amylase, glucosidase, maltase), glycolysis/gluconeogenesis (glycogen phosphorylase, fructose-1,6-bisphosphatase, aldolase, triose phosphate isomerase, glyceraldehyde-3-phosphate dehydrogenase, enolase), pentose cycle (phosphogluconate mutase, transketolase), fermentation (alcohol dehydrogenases, aldehyde dehydrogenase, formaldehyde dehydrogenase, acetyl CoA synthetase), TCA (isocitrate dehydrogenases, succinyl CoA synthetase, succinate dehydrogenase), electron transfer chain (NADH dehydrogenase, ubiquinol-cytochrome c oxidoreductase), and ATP synthesis (ATP synthase). In addition, a number of sequences in the class Triacylglycerols (18.8% DE sequences, the third most enriched class in PRC1a) may also fall within the broader category of ‘intermediary metabolism’ as they include sequences coding for synthesis and degradation of lipids centered around metabolism of acyl-CoA. The second most enriched class in PRC1a, Cryoprotection (22.5% DE sequences), also reflects well the metabolomics results showing differential regulation of ornithine concentrations. The best-predicted sequence of the class Cryoprotection is *ornithine decarboxylase 1*, and number of other DE sequences code for elements of ornithine metabolism (ornithine aminotransferase, pyridoxal-5-phosphate synthetase, glutamine synthetases, glutamate transporter). The fourth most enriched class in PRC1a, Detoxification (18.3% DE sequences) includes a number of sequences coding for enzymes responsible for coping with oxidative stress and redox signalling (catalase, superoxide dismutase, glutathione synthetase, glutathione S transferase, thioredoxine peroxidases, peroxiredoxins, methionine sulfoxide reductase). This class is also reflected in the metabolomics dataset in the differentially-regulated levels of cysteine, glycine, and glutamate (three components of glutathione).

There were only three sequences upregulated specifically in LN-treated larvae and not in the other two treatments (pattern PRC1b). Two of them, *hsp22* and *hsp26*, code for small heat shock proteins and *Atg8a* codes for an autophagy-specific protein. The small heat shock proteins stabilize early unfolding intermediates of aggregation-prone proteins, arising as a result of diverse stress conditions including cold shock^24,58^. The Hsp22 protein is localized in the mitochondrial matrix and its high level of expression in aging flies suggested a role in protection against oxidative stress^59^. Relatively high expression of different HSP-coding sequences was characteristically associated with recovery from all three cold stresses (see pattern PRC2a, enriched functional class Temperature, Fig. 4D). Thus, high expression of *hsp22* and *hsp26* specifically in the LN treatment may only confirm that protein denaturation was most severe in this particular treatment. The *Atg8a* codes for ubiquitin-like protein localizing to autophagosomes^60^. Autophagosomes serve to recycle large protein complexes and damaged organelles during insect metamorphosis, periods of starvation, or in response to injuries caused by oxidative stress, pathogenic infection, misfolded proteins, and hypoxia^60,61^. Transcriptional upregulation of *Atg8a*, and other autophagy-related genes, is characteristically observed just prior to cell death (induced developmentally or in response to stress) in various cell types in *D*. *melanogaster*^62,63^. High expression of *Atg8a* in the LN treatment is thus another marker of severe injury caused by cryopreservation stress.

## Conclusions

Contrary to our expectation, the metabolomic and transcriptomic profiles of recovery from cold stress differed between control and supercooled larvae (hypothesis I), were similar in supercooled and frozen larvae (hypothesis II), and differed between frozen and cryopreserved larvae (hypothesis III). PRC analysis draws two main division lines between: (i) control vs. all cold-stressed variants, and (ii) supercoooled and frozen (with high adult survivorship) vs. cryopreserved (exhibiting high proportion of delayed mortality) variants.

Despite the fact that diapausing, cold-acclimated *C*. *costata* larvae belong among the most cold-hardy animals on Earth^34^, our results suggest that they are injured by all three cold treatments. While most ( > 89.3%) of supercooled and frozen larvae were able to repair the injuries and successfully metamorphosed into adults, cryopreserved larvae exhibited relatively high (60.5%) levels of delayed mortality that occurred days or weeks after the end of cold stress. Repair processes required access to additional energy (larval CO_2_ production increased during recovery) exceeding the standard requirement by approximately 20%. Metabolomic profiling and custom microarray analyses provided hints on repair processes. In comparison to control larvae, all three cold stresses caused increased concentrations of anaerobic by-products (alanine, lactate, and succinate), metabolic markers of oxidative stress (cysteine), and amino-acid sinks for amino groups (glutamate, aspartate). In addition, 94 sequences were up-regulated while 24 sequences were down-regulated during recovery from all stresses. Among the upregulated sequences, the gene functional category ‘response to temperature stress’, including various heat shock proteins, was the most enriched. The metabolic perturbations observed specifically in cryopreserved larvae point toward impaired mitochondrial function and these larvae also failed to upregulate a set of 116 sequences that were up-regulated in supecooled and frozen larvae. These sequences covered functional categories of intermediary and energy metabolism, metabolism linked to potential cryoprotectants (proline, glutamine, trehalose), dealing with oxidative stress, and re-folding or removal of partially denatured proteins. In contrast, three sequences were specifically up-regulated in cryopreserved larvae: two small heat shock proteins *hsp22* and *hsp26*, and an autophagy-related gene *Atg8a*. Collectively, our results suggest that repair and disposal of damaged proteins is an important feature of recovery from cold stress in *C*. *costata* larvae.

## Methods

### Insects, diapause, cold-acclimation

Adult *Chymomyza costata* (Diptera: Drosophilidae) flies were originally collected in the wild in 1983, in Sapporo (43.1°N, 141.4°E), Hokkaido, Japan. Since then, the insect culture has been maintained in the laboratory on an artificial diet as described earlier^64^ in MIR154 incubators (Sanyo Electric, Osaka, Japan) under conditions promoting direct development, i.e. constant temperature of 18 °C and a long-day photoperiod (LD, Light:Dark phase, L16 h:D8 h). Diapause was induced in experimental cohorts of larvae by rearing them at a constant temperature of 18 °C and a short-day photoperiod (SD, L12 h:D12 h). Under these conditions, all individuals respond reliably to photoperiodic signal and enter into diapause as fully grown 3rd instar larvae^65–68^. Next, diapausing larvae were cold acclimated by transferring them to 11 °C and constant darkness at 6 weeks of age and, two weeks later, transferring them to 4 °C for another 4 weeks. This gradual, 6-week cold acclimation (SDA) dramatically enhances freeze tolerance such that frozen larvae survive when cryopreserved in liquid nitrogen (LN_2_)^34^.

### Exposure to cold stress, survival

Twelve-week-old, cold-acclimated, diapausing larvae (SDA) were either directly sampled for analyses (control, C) or were exposed to one of three different cold stresses in a programmable Ministat 240 cooling circulator (Huber, Offenburg, Germany): supercooling (S) to −10 °C; freezing (F) to −30 °C; or cryopreservation (LN) in LN_2_ (for schematic depiction of the experimental design, see Fig. S1). Larvae destined for cold exposure were separated from the larval diet by washing in ice-cold water and groups of approximately 20 larvae were placed in between two layers of cellulose (75 mg) that were moistened with 300 µl of distilled water. The moist cellulose ‘ball’ with larvae inside was inserted into a plastic tube (diameter, 1 cm; length, 5 cm), and the tube placed in the cooling circulator at the start of temperature program. The temperature inside the cellulose ball was continuously monitored in control tubes (containing no larvae) with K-type thermocouples connected to a PicoLog TC-08 datalogger (Pico Technology, St. Neots, United Kingdom). To ensure *supercooling* conditions for the S treatment, a 50% glycerol solution was used to moisten the cellulose ball instead of distilled water. Glycerol prevents the occurrence of spontaenous freezing of water inside the ball and we verified in preliminary experiments that glycerol has no effect on larval survival (neither positive nor negative). To ensure *freezing* conditions in the F tyreatment, a small ice crystal was added to the surface of the moist cellulose (at the start of the temperature program), which results in immediate freezing of water inside the ball and stimulates ice penetration and inoculative freezing of larval body fluids. To assess *cryopreservation*, the larvae gradually frozen to −30 °C were plunged in LN_2_ for one hour and the returned back to −30 °C. The temperature programs were described in detail previously^34,35^, and are schematically presented on Figure S1. At the end of program, the unpacked cellulose balls were transferred on fresh standard diet in a tube maintained at constant 18 °C. Alive/dead larvae were scored after 12 h recovery. All living larvae were maintained at 18 °C for a subsequent 6 weeks and succesful pupariation and emergence of adult flies were scored as ultimate criterions of survival. In addition, we measured the fresh mass (FM) and dry mass (DM, after three days of drying at 60 °C) in 40 survivor adults (20 males, 20 females) from the control and cold treatments. Exact numbers of larvae used for each specific experiment are shown in Results.

Although we sampled only the apparently living specimens (those moving spontaneously and crawling in a coordinated manner), we cannot exclude the possibility that an unknown proportion of sampled specimens were in fact doomed and, therefore, displaying metabolomic and transcriptomic changes leading to death rather than to repair of cold injury and successful recovery. This drawback is inherently present in all studies where only a fraction of individuals survive the treatment.

### Potassium ions concentration in hemolymph and CO2 production

In order to estimate the rate of recovery from cold stress in control and cold-exposed larvae, we analyzed the concentration of potassium ions [K^+^] in the hemolymph, and the production of CO_2_. Hemolymph [K^+^] was measured using an MI-442 K^+^ Ion Microelectrode in combination with a reference electrode MI-402 (both from Microelectrodes Inc., Bedford, NH, USA). A sample of hemolymph was collected from a pool of 10–20 larvae (to reach ca. 3 µl in total) into calibrated micro-capillary tube (Broomall, PA, USA). Exactly 2.5 µl of hemolymph was then diluted 3 times with 5 µl of deionized water in order to obtain sufficient volume for microelectrodes (7.5 µl). Three to six biological replicates (pools of 10–20 larvae) were measured as soon as possible after the end of cold stress treatment (first sampling) and one hour after the end of cold stress treatment (second sampling). The manipulation of 10–20 larvae (unwrapping the cellulose ball, tearing, and collecting the hemolymph into the capillary) took approximately 15 min at room temperature. Voltage was measured using pH/mV Hand-Held Meter pH 330 (WTW, Weilheim, Germany) and converted to [K^+^] using a semilog line regression calibration curve. The calibration samples (1 mM, 10 mM, 100 mM KCl solution) were measured just prior to measuring the hemolymph samples on every occasion. The one-way ANOVA was used to analyze whether there is any influence of the treatment on [K^+^] and the post-hoc Bonferroni’s multiple comparisons tests were applied to find the differences among particular treatments. Data were initially tested for normality (Kolmogorov-Smirno test) and homoscedascity (Bartlett’s test) before subjecting them to ANOVA. Unpaired two-tailed *t*-tests were used to assess the differences in [K^+^] between two specific treatments. The *F* tests were applied first to verify that variances of the two menas do not significantly differ. These statistical calculations were performed using Prism6 (GraphPad software, San Diego, CA, USA).

The production of CO_2_ was measured using the respirometry analysis system MFC-2/TR-SS3/MUX (Sable Systems International, Las Vegas, NV, USA) equipped with LI 7000 CO_2_/H_2_O analyzer (LI-COR Biosciences, Bad Homburg, Germany). Six groups of 10 larvae of known FM (for each experimental variant) were placed in six glass tubes (volume 22 ml) on 1 g of larval diet that was sterilized by application of PenStrep (Sigma-Aldrich, Saint Luis, MO, USA) in concentrations 100 U peniciline and 0.1 mg streptomycine per 10 g diet. Two another tubes served as blank controls (an empty glass tube, and a glass tube containing PenStrep-treated diet only). All tubes were placed inside the MIR154 incubator set to a constant temperature of 18 °C and a short-day photoperiod (SD) where Zeitgeber time 0 was set to the moment when the cold exposure finished. The manipulations following completion of the cold exposure program (unwrapping the cellulose ball, counting larvae, weighing FM, and transferring larvae to glass tubes) took approximately 30 min, and the analysis of CO_2_ production started immediately after. Larvae lived inside tubes for 72 h and their CO_2_ production was measured in 30 min intervals. During each interval, the system was ‘closed’ for 1,575 sec and then flushed for 225 sec using 75 ml of CO_2_-devoid air from a tank (Linde Gas, Praha, Czech Republic). The CO_2_ was removed using CO_2_-absorbent soda lime (Elemental Microanalysis, Okehampton, UK). We used the ExpeData software tool (v.1.2.02, Sable Systems International) to process the CO_2_ data. After 72 h, surviving larvae were weighed a second time and the two FM records were used to calculate the CO_2_ in µl/h.mg^−1^ FM assuming a linear increase in FM during the three days (survival was typically 100%; if not, the FM of dead larvae was subtracted from calculations for second half of the three day period). The differences in CO_2_ production among treatments were assessed using ANOVA followed by Bonferroni’s test as described above (Prism6). In addition, we searched for daily rhythmic pattern in CO_2_ production data by visual inspection of the data and also using two statistical methods, a shareware program CircVawe v.1.4 (Roelof A. Hut, University of Groningen, Netherlands, http://www.rug.nl/fwn/onderzoek/programmas/biologie/chronobiologie/downloads/index), and a Lomb-Scargle *P*_x_ periodogram^69,70^. The raw data for CO_2_ production and results of statistical analysis are presented in Dataset S1.

### Targeted metabolomics

Pools of five larvae (in four biological replicates) were sampled at four time points of recovery from cold stress: 0, 12, 36, and 72 h (see Fig. S1). The first sample (time 0) was taken at low temperature (4 °C in control; 5 °C, in cold treatments). The larvae were weighed for FM, plunged into LN_2_, and stored at −80 °C until analysis. The pools of larvae were homogenized in 400 μl of a methanol:acetonitrile:water mixture (volumetric ratio, 2:2:1) containing internal standards (*p*-fluoro-DL-phenylalanine, methyl α-D-glucopyranoside; both at a final concentration of 200 nmol.ml^−1^; both from Sigma-Aldrich, Saint Luis, MI, USA). The TissueLyser LT (Qiagen, Hilden, Germany) was set to 50 Hz for 5 min (with a rotor pre-chilled to −20 °C). Homogenization was repeated twice and two supernatants stemming from centrifugation at 20,000 g/5 min/4 °C were combined. The extracts were subjected to targeted analysis of major metabolites using a combination of mass spectrometry-based analytical methods described previously^56^.

Low-molecular-weight sugars and polyols were determined after *o*-methyloxime trimethylsilyl derivatization using a gas chromatograph (GC) with flame ionization detector GC-FID-2014 equipped with AOC-20i autosampler (both from Shimadzu Corporation, Kyoto, Japan). Profiling of acidic metabolites was done after treatment with ethyl chloroformate under pyridine catalysis and simultaneous extraction in chloroform^71^. The analyses were conducted using Trace 1300 GC combined with single quadrupole mass spectrometry (ISQ) (both from Thermo Fisher Scientific, San Jose, CA, USA) and a liquid chromatograph Dionex Ultimate 3000 coupled with high resolution mass spectrometer Q Exactive Plus (all from Thermo Fisher Scientific). All metabolites were identified against relevant standards and subjected to quantitative analysis using a standard calibration curve method. All standards were purchased from Sigma-Aldrich. The analytical methods were validated by simultaneously running blanks (no larvae in the sample), standard biological quality-control samples (the periodic analysis of a standardized larval sample – the pool of all samples), and quality-control mixtures of amino acids (AAS18, Sigma Aldrich).

The results of targeted metabolomics (as summarized in Dataset S2, Excel sheet: Raw data) were subjected to statistical analysis based on constrained linear ordination methods using Canoco software (v. 5.04)^72^. Concentrations of metabolites were first log-transformed (using a ln(100*y + *1)* formula) and then centered and standardized. We used a specialized multivariate method called principal response curves (PRC) which focuses on time-dependent treatment effects on multivariate response data^73^. Each principal component (corresponding to first and higher axes of the underlying partial RDA) is plotted against time, yielding one PRC curve for each experimental variant. In addition, the temporal pattern of metabolomic changes in the control variant (C) was set as a reference value (0) and the temporal patterns of the treatments (S, F, LN) represent differences from the control.

### Custom microarray analysis

Transcriptomic profiling was based on 1,124 candidate sequences arbitrarily selected from published Illumina RNAseq database that contains 21,327 putative mRNA transcripts of *C*. *costata* (ArrayExpress accession E-MTAB-3620). The sequences were annotated and manually classified into 15 functional categories based on GO terms, InterPro, FlyBase, KEGG and other descriptions^74^. The selected genes broadly cover major structures and processes known, or suggested, to be linked to insect diapause and cold tolerance. The methods for custom microarray production were described previously^68^. We added 79 new sequences, putatively involved in DNA repair and processing of unfolded proteins, to the second generation of custom microarray (Cos2 microarray). The complete list of Cos2 microarray sequences is presented in supplementary material (Dataset S3, Excel sheet: Sequences and PRC analysis).

Pools of five larvae were sampled at four time points of recovery from cold stress: 0, 12, 36, and 72 h (see Fig. S1). There were two levels of replication: technical triplicates of each spot on the microarray (1, 2, 3), and biological replication of triplicate samples for each treatment (separate pools of larvae: a, b, c). The first sample (time 0) was taken at low temperature (4 °C in control; 5 °C, in cold treatments). Sampled larvae were stored in RiboZol RNA Extraction Reagent (Amresco, Solon, OH, USA) at −80 °C prior to processing. All details of sample processing were the same as published earlier^68^. Briefly: Total RNA was isolated using RiboZol and treated with DNase I (Ambion, Life Technologies, Foster City, CA, USA). For the first strand cDNA synthesis, we used the Oligo(dT)_23_ anchored primer (Sigma-Aldrich) and Superscript III (Invitrogen, Carlsbad, CA, USA). The second DNA strand was synthesized using DNA polymerase I and treated with RNaseH (both Invitrogen). Next, double strand DNA was purified using Wizard SV Gel and PCR Clean-up System (Promega, Madison, WI, USA). The double strand DNA was labelled with Cyanine-5-dCTP dye (Cy5, PerkinElmer, Waltham, MA, USA) using Klenow fragment polymerase and the BioPrime DNA Labelling System (Invitrogen). After removing the unincorporated dye (Illustra AutoSeq G-50 Dye Terminator Removal Kit, GE Healthcare, Little Chalfont, UK) and adding a blocking agent (sonicated salmon sperm DNA, Invitrogen), the labelled double strand DNA was used for hybridization onto microarrays. An automatic Hybridization Station HS4800 Pro (Tecan, Mannedorf, Switzerland) was used to perform all the hybridization steps (washing the station, pre-hybridization, hybridization, rinsing and drying). All microarrays were scanned immediately upon completion of the hybridization procedure using the ScanArray G_x_ Microarray Scanner (PerkinElmer) at a resolution of 5 µm and the fluorescence was quantified using ScanArray Express v. 4.0.0.0004 software (Perkin Elmer). Fluorescence values were normalized between arrays using Quantile normalization^75^. All log2-normalized fluorescence values are summarized in Dataset S3 (Excel sheet: Raw data). Next, mean log2-normalized fluorescence values were calculated by averaging the spot technical triplicates and the resulting dataset was used for principal response curves (PRC) analysis (Canoco 5) similarly as described for metabolites.

We selected four sequences for technical validation of microarray analysis results by quantitative real time PCR (qPCR) using a CFX96 PCR cycler (BioRad, Philadelphia, PA, USA). The relative mRNA transcript abundance of selected target sequences was measured in the aliquots of the same total RNA as subjected previously to microarray analysis. The total RNA treated with DNase I (Ambion) was used for the first strand cDNA synthesis primed with Oligo(dT)_23_ anchored primer (Sigma-Aldrich) using Superscript III (Invitrogen). The cDNA products (20 µL) were diluted 25 times with sterile water. PCR reactions (total volume of 20 µL) contained 5 µL of diluted cDNA template, LA Hot Start Plain Master Mix (TopBio, Vestec, Czech Republic), and were primed with a pair of gene-specific oligonucleotide primers (Table S1, Dataset S3, Excel sheet: qRT-PCR validation), each supplied in a final concentration of 400 nM. Cycling parameters were 3 min at 95 °C followed by 40 cycles of 94 °C for 15 s, 60 °C for 30 s and 72 °C for 30 s. Analysis of melt curves verified that only one product was amplified in each reaction. In addition, we checked the size of the PCR products for each gene by electrophoresis on 2% agarose gel in selected samples. Emission of a fluorescent signal resulting from SYBR Green binding to double-stranded DNA PCR products was detected with increasing PCR cycle number. Threshold cycle (*C*_T_) for each sample was automatically calculated using the algorithm built in the CFX96 PCR light cycler software. We used two genes coding for Ribosomal proteins, *RpL19* and *RpL32* as endogenous reference standards for relative quantification of the target transcript levels^68^. Each sample was run in duplicate (two technical replicates) of which the mean was taken for calculation. Relative ratios of the target mRNA levels (*C*_T_) to geometric mean of the levels (*C*_T_) of two reference gene mRNAs were calculated as dd*C*_T_^76^.

## Electronic supplementary material

Figure S1

Dataset S1

Dataset S2

Dataset S3
