# Supplementary material for: Recovery from supercooling, freezing, and cryopreservation stress in larvae of the drosophilid fly, Chymomyza costata
Source: Sci Rep. 2018 Mar 13;8:4414. doi: 10.1038/s41598-018-22757-0 (PMC5849770; doi:10.1038/s41598-018-22757-0)
Supplement: Supplementary file 1 — Figure S1 [file 41598_2018_22757_MOESM1_ESM.docx]

**Recovery from supercooling, freezing, and cryopreservation stress in larvae of the drosophilid fly, *Chymomyza costata*.**

Tomáš Štětina, Petr Hůla, Martin Moos, Petr Šimek, Petr Šmilauer, Vladimír Košťál

**Supplementary Information**


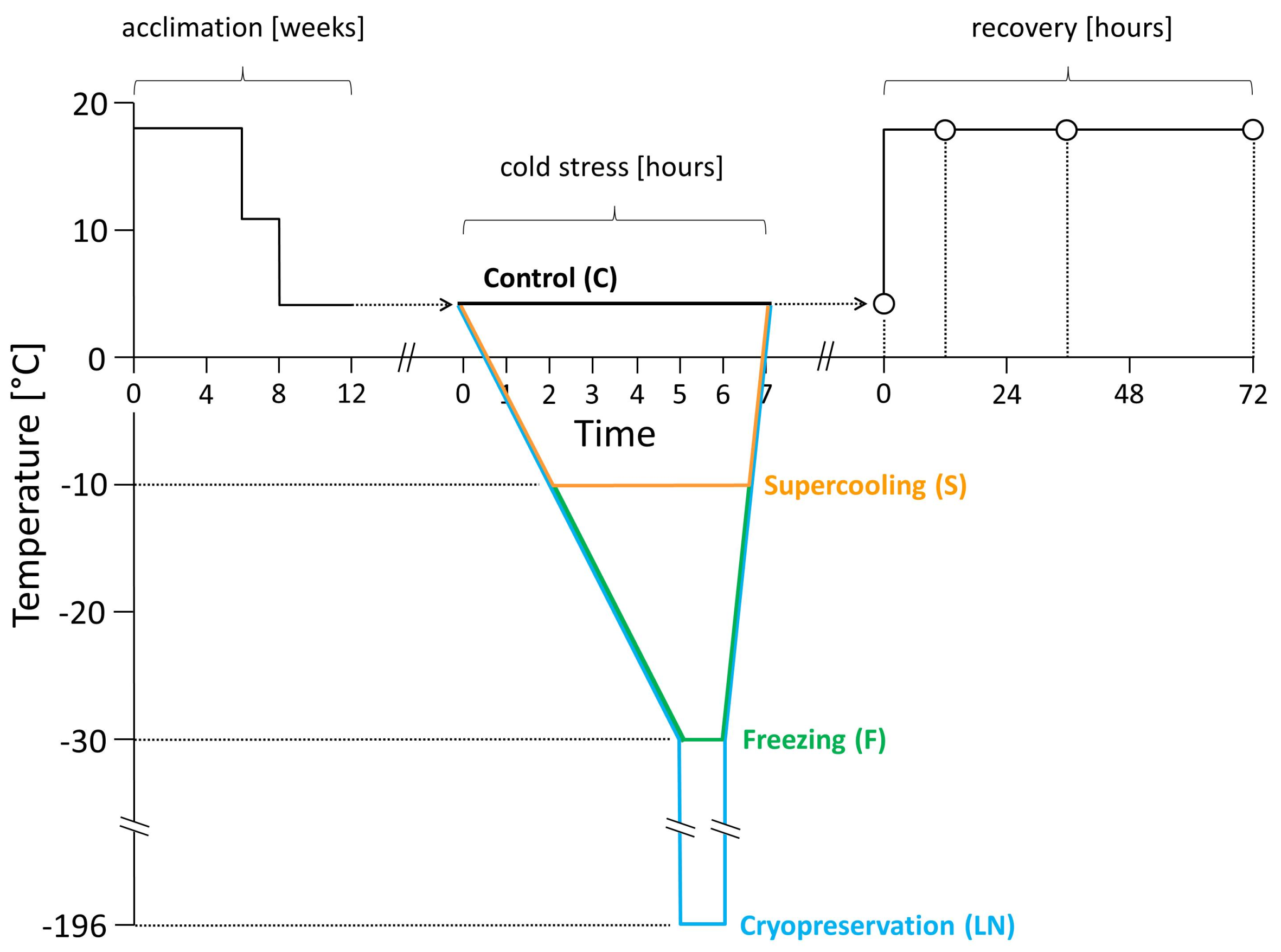


**Figure S1.** **Schematic depiction of experimental design used to analyze the metabolomic and transcriptomic responses in control and cold-stressed larvae of Chymomyza costata.**

Most of the experimental larvae were diapausing, cold-acclimated (SDA) meaning that they were reared under short days and 18°C for 6 weeks and, subsequently, gradually cold acclimated at 11°C for 2 weeks followed by 4 weeks at 4°C. Next, the SDA larvae were either directly transferred to 18°C (control, C) or were cold-stressed. Three levels of cold stress were tested: (S), supercooling to -10**°C; (F), freezing at -30°C; or (LN), or cryopreservation in liquid nitrogen. After the cold stress, the larvae were transferred to 18°C for recovery. During the recovery from cold stress, the concentrations of hemolymph potassium, CO_2_ production, and profiles the metabolome and transcriptome were assessed (at times 0, 12, 36 and 72 h of recovery).**

Two other acclimation variants (LD and SD) were specifically used as controls for analysis of [K^+^]. The LD larvae came from continuous culture maintained under conditions promoting direct development, *i.e.* constant temperature of 18°C and a long-day photoperiod. The SD larvae are 6-week old larvae reared under short days and 18°C, i.e. early diapausing, warm acclimated.
